# Robotic Retroperitoneal Lymph Node Dissection for Testicular Cancer—First Experience and Learning Curve of a Single Surgeon

**DOI:** 10.3390/cancers17091476

**Published:** 2025-04-27

**Authors:** Markus Angerer, Christian Wülfing, Klaus-Peter Dieckmann

**Affiliations:** Department of Urology, Asklepios Klinik Altona, Paul-Ehrlich-Strasse 1, 22763 Hamburg, Germany

**Keywords:** testicular cancer, retroperitoneal lymph node dissection, robotic surgery, learning curve

## Abstract

**Background/Objectives:** Retroperitoneal lymph node dissection (RPLND) plays a crucial role in the staging and treatment of testicular cancer and is often mandatory. RPLND is associated with a high risk of morbidity. The use of minimally invasive techniques has significantly increased the number of robotic procedures performed over the last few years. This study aimed to analyze the perioperative and postoperative outcomes and trends of an increasing number of surgeries performed. **Materials and Methods:** We retrospectively analyzed 30 robotic RPLNDs (R-RPLNDs) performed at our testicular cancer center between 2020 and 2024. Logistic regression analyses were used to analyze the independent variables of operative time (OT), hospital stay (HS), estimated blood loss, lymph node yield, and complications according to the Clavien–Dindo classification system. The independent predictors included case number, clinical stage, post-chemotherapy status, preoperative retroperitoneal tumor mass, and body mass index. Furthermore, the patients were categorized into three groups: group A (cases 1–10), group B (cases 11–20), and group C (cases 21–30). A Kruskal–Wallis test was performed to assess differences among the groups concerning OT, HS, and lymph node yield. **Results:** OT significantly decreased with an increasing number of cases (*p* < 0.001), and HS was significantly affected by overall complications (*p* = 0.0006). There were two major perioperative complications (6.6%). No factors predicted overall complications or Clavien–Dindo grades I-II or III–V. The Kruskal–Wallis test showed a significant difference (*p* < 0.05) in OT and HS for group C. **Conclusions:** R-RPLND for GCTs demonstrates a clear learning curve, with significant improvements in OT, HS, and complication rates as surgeons gain experience. Overall, the low complication rates for R-RPLND did not indicate predictive factors for perioperative or postoperative complications. RPLND presents promising potential as a safe and effective treatment for GCTs, especially when performed by experienced surgeons in specialized centers.

## Simple Summary

Retroperitoneal lymph node dissection (RPLND) plays a crucial role in the staging and treatment of testicular cancer. This study explores how using robotic surgery for this procedure can improve patient outcomes. We reviewed the first 30 RPLND cases performed at our center to see how surgical results changed as the surgeons gained experience. This study found that surgeries became quicker and hospital stays shorter as more procedures were performed, suggesting that surgeon experience plays a key role. These findings could help improve surgical care for testicular cancer by supporting the use of robotic surgery in experienced hands, potentially making the procedure safer and more effective over time.

## 1. Introduction

Retroperitoneal lymph node dissection (RPLND) was once a cornerstone of the multimodal treatment for metastatic germ cell tumors (GCTs) of the testis, following its technical refinement in the 1960s and 1970s. The primary mode of metastatic spread in testicular GCTs was established around the turn of the 19th and 20th centuries [1]. Its significance diminished with the introduction of cisplatin-based chemotherapy. However, due to growing concerns over the severe long-term side effects of chemotherapy, RPLND is recently regaining importance. The most frequent indication for RPLND now occurs in the post-chemotherapy setting. Additionally, RPLND is increasingly used for primary cases with low-volume lymphadenopathy where tumor markers are not elevated. Open RPLND (O-RPLND) has thus far been the standard procedure, representing a complex and potentially morbid treatment modality, particularly in the post-chemotherapy setting. Almost half of patients with NSGCTs have metastatic disease at the time of initial presentation [2]. The recognized risks associated with RPLND include intraoperative hemorrhage from large vessel laceration, damage to neighboring organs, postoperative ileus, chylous ascites, and retrograde ejaculation. The reported complication rate in the primary setting is approximately 10–20% [3].

Over the past two decades, minimally invasive approaches have been reported to decrease procedure-related morbidity. The first robot-assisted retroperitoneal lymph node dissection (R-RPLND) was performed in 2006 at Geisinger Medical Center in Pennsylvania [3]. In several studies, R-RPLND demonstrated promising potential as a safe treatment for germ cell tumors (GCTs), with comparable oncologic efficacy to open RPLND, especially when conducted by experienced surgeons in specialized centers. The key advantages of R-RPLND over open RPLND include shorter operating time, reduced postoperative pain, and decreased hospital stay (HS), which facilitate quicker recovery. In the post-chemotherapy setting, R-RPLND also appears feasible, with an acceptable complication rate and low conversion rates to open surgery [4].

With the increasing worldwide availability of robotic surgery facilities, increased attention has been directed toward the learning curve associated with this novel technological approach. The aim of this study was to evaluate the first series of R-RPLNDs performed in our institution, analyze associations between clinico-oncological features and surgical results, and, particularly, examine the learning curve of a single surgeon. We hypothesized that the complications rate would be associated with oncological characteristics and that increasing experience would translate into decreases in operation time (OT), estimated blood loss (EBL), and the frequency of operative complications.

## 2. Methods

We retrospectively reviewed the first consecutive 30 R-RPLNDs performed for GCTs at our center from 2020 to 2024. The cases were identified through the institutional electronic patient record system. Patient selection for robotic RPLND was based on the extent of the retroperitoneal tumor mass. Thus, cases with a >5 cm tumor size and those with more than 2 templates to be resected were excluded and subjected to open standard RPLND.

**Figure 1:**
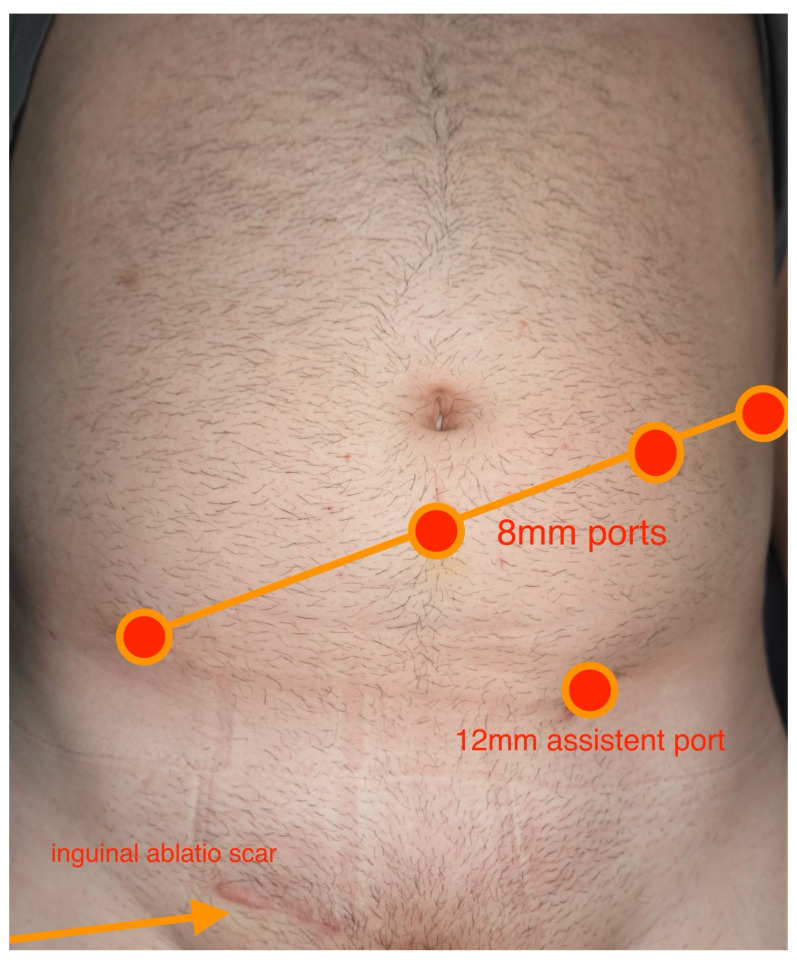
Overview of intraoperative port placement.

R-RPLND was performed using the DaVinci X/Xi System through a transperitoneal approach with the patient in the Trendelenburg position and the robot docked over the patient’s head. The exact port placement depended on the location of the target area. In general, a four-port oblique line placement was used. The camera port (8 mm) was placed in a midline position, 4 cm caudal to the umbilicus, and three additional ports (8 mm) were placed in an oblique line, including an assistant port (12 mm) (Figure 1). The surgical resection was always template-based. The boundary of the right unilateral template was defined by the right renal artery cranially, bifurcation of the right common iliac artery caudally, the right ureter laterally, and pre-aortic nodes in the medial direction. As for left unilateral template, the boundary was renal arteries in the superior direction, the bifurcation of the common iliac arteries in the inferior direction, the left ureter in the lateral direction, and the inferior vena cava in the medial direction. No re-docking was needed in any of the cases [5]. All operations were performed by one single surgeon (CW).

Patient demographics (age, BMI) and tumor characteristics, including the pathology of the primary tumor, were recorded along with the preoperative staging measurement of the largest transverse diameter of the retroperitoneal nodes, clinical stage, IGCCCG risk-group classification, and previous chemotherapy regimens. Additionally, the surgical field (unilateral/bilateral), OT, EBL, HS, final pathology of the resected specimen, and lymph node count were documented. OT was defined as the time interval from the first incision to last stitch. Complications within 90 days following surgery were graded according to the Clavien–Dindo classification. Grade I–II complications were defined as minor and Grade IIIa–V as major. The median time of follow-up was noted, as well as the occurrence of relapse or other untoward events. For statistical analysis, the patients were categorized into three groups: group A (consecutive cases 1–10), group B (cases 11–20), and group C (cases 21–30). The three groups widely mirrored three consecutive periods of time during the course of the 5-year study period. To ensure consistent data sets for statistical comparisons, three groups with identical patient numbers were created.

We determined the median values and interquartile ranges (IQRs) of OT, HS, EBL, lymph node count, as well as complications according to the Clavien–Dindo classification system for the entire patient population and for each of the three patient categories. The results of the three patient categories were compared to one another using the Kruskal–Wallis test. Logistic regression analyses were used to analyze independent variables such as case number (CN), clinical stage (CS), chemotherapy status, preoperative retroperitoneal mass size, final histology, and body mass index (BMI). The comparisons between the groups involved both univariate and multivariate analyses. Patients with missing data were excluded from the corresponding subanalyses. Statistical significance was set at *p* < 0.05. R-Studio (Posit Software, PBC formerly RStudio, PBC, Version 2024.09.1+394, 2024, Boston, MA, USA) was used for analysis, and DATAtab (DATAtab Team 2024, Online Statistics Calculator. DATAtab e.U. Graz, Austria) was used for graphical analysis.

All study activities were conducted in compliance with the Helsinki Declaration of the World Medical Association as amended by the 64th General Assembly (2013). Ethical approval was provided by the Ethikkommission der Ärztekammer Hamburg (PV7288, 2 March 2020).

## 3. Results

The first 30 patients with GCTs undergoing R-RPLND in our institution were retrospectively included in this study. Their clinical details are summarized in Table 1. On average, the higher median patient ages in groups B and C relate to the increasing referrals of seminoma patients to primary lymph node dissection in more recent years.

Among the entire patient population (*n* = 30), the median OT (first incision to last stitch) was 186.5 min, the median EBL was 100 mL, and the median length of hospital stay was 6.5 days. The median size of retroperitoneal lymph nodes on preoperative CT (largest axial lymph node diameter) was 2 cm, the median lymph node yield was *n* = 13, and the median number of GCT-harboring positive lymph nodes was two. There were two (6.6%) major perioperative complications, both in pcRPLNDs. One (3.3%) was a conversion to an open procedure secondary to hemorrhage from aortic laceration rescued by aortic grafting (Grade IIIb). The other was a hemorrhage from vena cava laceration, which was oversewn without conversion (Grade IIIa). Postoperatively, seven (23.3%) Grade II complications were noticed: one postoperative anemia requiring blood transfusion, three patients with prolonged lymphorrea (all primary R-RPLND), two patients with chylous ascites (one primary and one pcRPLND), and one transient paresis of the legs. No patient needed extra percutaneous drainage. There were no Clavien–Dindo Grade 4 or 5 complications among all the R-RPLND cases.

A comparison of the results among the three patient groups is presented in Table 2 and Table 3.

**Figure 2:**
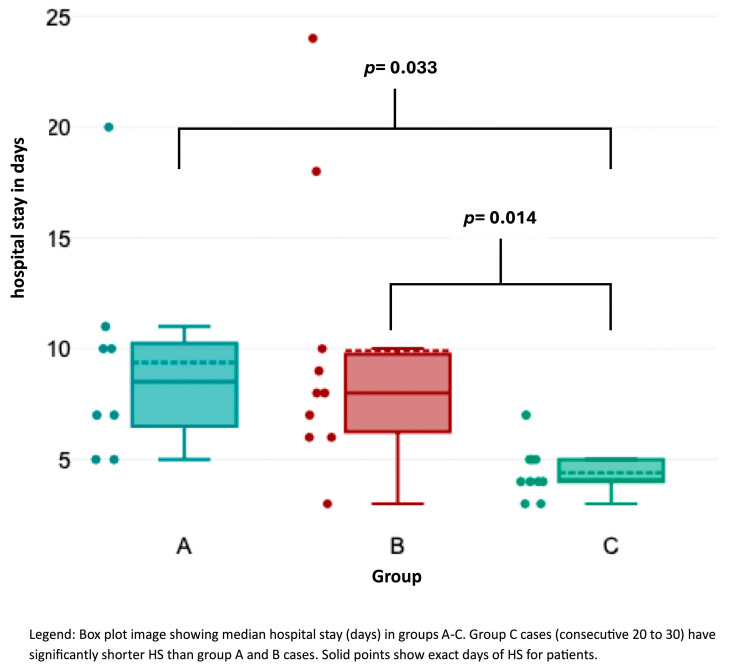
Association of hospital stay with consecutive case number.

**Figure 3:**
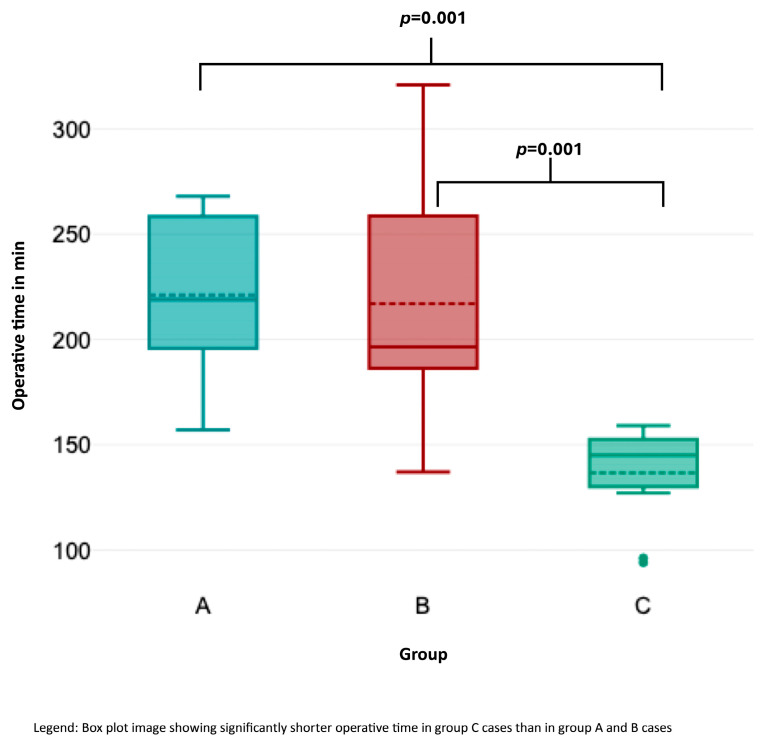
Association of operative time with consecutive case number.

**Figure 4:**
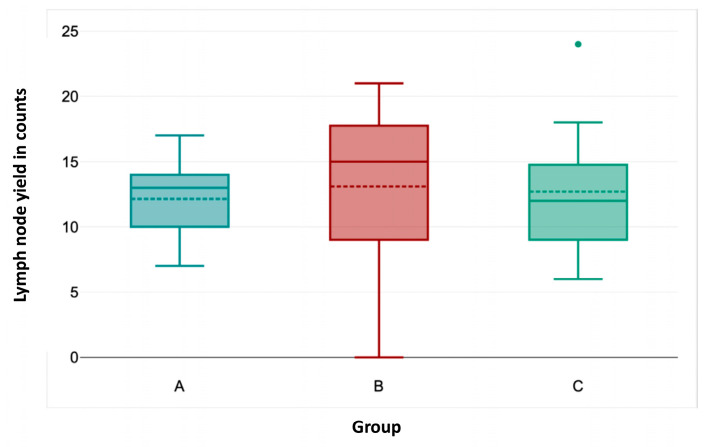
Association of lymph node yield with consecutive case number.

OT (Figure 2) was significantly shorter in group C than in groups A and B (Kruskal–Wallis test, *p* < 0.001). Likewise, HS (Figure 3) was significantly shorter in group C compared to the other groups (*p* = 0.033 for group A–C; *p* = 0.014 for groups B–C). Lymph node yield was not significantly different in number among groups A to C (Figure 4).

By contrast, no significant differences among the groups was seen for EBL (*p* = 0.461), lymph node yield (*p* = 0.942), positive lymph nodes (*p* = 0.5275), preoperative mass size (*p* = 0.963), and preoperative LDH, AFP, and ß-HCG (*p* = 0.405, *p* = 0.370, and *p* = 0.735, respectively). Furthermore, groups A to C were not statistically different regarding the relative proportion of post-chemotherapy procedures, clinical stages, and prior abdominal operations (*p* = 0.877, *p* = 0.231, and *p* = 0.757, respectively).

The significantly shorter OT with increasing consecutive case numbers was confirmed in our multivariate analysis (Table 4, *p* = 0.01). HS was significantly affected by overall complications (*p* = 0.0006), but only by low-grade complications (*p* = 0.0001), not by high-grade complications (*p* = 0.6). In multivariate regression analysis, HS was significantly longer with low-grade complications (*p* = 0.003) and high-grade complications (*p* = 0.02). In addition, uni- and multivariate logistic regression analysis regarding complications did not disclose any predicting factor for overall, low-grade, or high-grade complications, respectively. Remarkably, higher lymph node and positive lymph node counts did not impact the frequency of chylous ascites (Clavien Dindo II). The results for the uni- and multivariate analyses are shown in Table 5, Table 6 and Table 7.

### Pathological and Oncological Outcomes

In the entire patient population, the final pathology of the R-RPLND specimens revealed viable tumors in 24 (80%) patients, including teratoma in 9 (30%), embryonal carcinoma in 9 (30%), a primitive neuroendocrine tumor (PNET) in 1 (3.3%), seminoma in 4 (13.3%), and a mixed histology of seminoma and embryonal carcinoma in 1 (3.3%). Five patients with pc-RPLND (20%) had fibrosis/necrosis, histologically. One patient with suspected lymph node metastasis of seminoma (3.3%) had no malignancy detected (pN0). The median follow-up was 16 months. Eleven (35%) patients received adjuvant chemotherapy.

Two (6.4%) patients experienced recurrence following R-RPLND. One patient developed an extensively bulky retroperitoneal disease 1 month after R-RPLND, and he was subsequently referred to high-dose chemotherapy. One relapsed with teratoma in the retrocrural space and at the cranial border of the previous R-RPLND template 13 months postoperatively. He was treated with open redo-RPLND.

Logistic regression analysis for relapse showed that no factor (case number, post-chemotherapy, CS, preoperative mass size, lymph node count, positive lymph nodes, or complications) has an impact on relapse rates in the entire population (*n* = 30). Also, group ranking (categories A to C) had no significant impact on relapse rate (Kruskal–Wallis test, *p* = 0.126).

## 4. Discussion

Our study evaluates the learning curve for R-RPLND in the primary and post-chemotherapy testicular cancer setting with regard to various peri- and postoperative parameters. To the best of our knowledge, this study constitutes the first assessment of distinct groups of case numbers (in chronological sequence) within the learning curve.

The crucial result of the present study is that robotic RPLND is a feasible procedure with a low complication rate and acceptable oncological outcome, but it requires a learning curve for each surgeon embarking on this procedure. Multiple studies have consistently demonstrated that robotic RPLND outperforms traditional approaches by significantly reducing perioperative complications [6,7].

Our findings clearly indicate a significant reduction in OT and HS with increasing surgeon’s experience. One other study found that OT is predicted to decrease by one hour after performing surgeries for 44 cases, highlighting the importance of experience in improving efficiency [8]. But OT is also considerably influenced by the individual surgeon’s experience and talent, with some surgeons achieving faster reductions in time than others [8]. Anceschi et al. suggest improvements occur after 12 months from the beginning of the learning course with structured long-term training programs for radical prostatectomy [9].

An important aspect of the learning curve is that increasing case numbers were associated with fewer overall complications. The rate of major complications (Clavien Dindo III, IV) is 6.6% in our series. Previous reports suggested significantly lower overall complication rates in the R-RPLND group than in the O-RPLND group but similar major (Grade ≥ III) complication rates in approximately 5–11% of cases, respectively [5,6,10,11,12]. Our complication rate also compares well with the complication rate of 14.4% reported in a German series of 146 pcRPLNDs and with an 8.8% complication rate in primary O-RPLND for 35 cases [13]. Nason et al. reported a low complication rate (3.7% minor, 11.1% major) for R-RPLND [3]. In 2020, a literature survey revealed that Clavien Dindo II/IV complication rates in pc-RPLND were as high as 23%, with the lowest rates found in institutions with high case loads [13]. Our study, which included both pcRPLND and primary resections, demonstrated complication rates comparable to those of the O-RPLND series. Reported overall complication rates for O-RPLND ranged from 16.6% to 37.9%, while major complications occurred in 8.3% of cases [4,7,14]. In our study, the most common complication was lymph leakage (a minor complication). As this particular complication can usually be resolved with conservative measures within a few days, no major consequences from the operation are expected in the long term. Consequently, patient safety is not compromised during a surgeon’s early case sequence for this procedure [8].

The total lymph node count is an important performance measure in oncologic surgery [8]. The number of lymph nodes retrieved during R-RPLND was comparable to or higher than that retrieved during L-RPLND, indicating effective oncological control [6,10]. Total lymph node count has also been shown to be an independent predictor of recurrence in the post-chemotherapy RPLND setting. Our series has shown that the total lymph node count was higher with increased BMI and CS III, and it was also significantly higher in larger preoperative mass size and CS III, as found in the multivariate analysis. The positive lymph node count was only influenced by the post-chemotherapy setting in the univariate analysis. However, the lymph node count did not change within the case numbers. This suggests that lymph node count is not affected by the earlier stages of the learning curve [8,15].

As shown in the present study, the occurrence of relapses was not associated with any particular risk factor, and it is also not affected by the learning curve, proving a safe short-term oncological outcome. Notably, several other studies revealed no differences in recurrence rates between robotic-assisted and standard open RPNLD [4,16,17,18].

The introduction of robotic surgery has revolutionized the field of urological oncology, with several studies demonstrating many benefits of robot-assisted approaches in general urology compared to open surgery [19,20,21]. The surgical platform for robot-assisted surgery has enabled many surgeons to perform minimally invasive surgery, which is popular and offers prospective benefits to patients, such as shorter HS, earlier recovery, less pain, and operational benefits to surgeons [22].

After the first R-RPLND in 2006, several studies have proved its non-inferiority to standard O-RPLND regarding oncological outcomes [3,23,24,25,26]. Meanwhile, R-RPLND has demonstrated its superiority over O-RPLND in perioperative outcomes, especially with respect to OT, EBL, and HS. Several recent reports have considered RPLND a de-escalating approach aiming to maintain the traditional excellent oncologic result while minimizing treatment burden and toxicity [4,24,27]. In fact, R-RPLND involves the potential of a paradigm shift in the management of testicular cancer, particularly in patients with clinical stage I–II nonseminomatous germ cell tumors (NSGCTs) requiring surgical intervention [11,28]. Nevertheless, the goals of any innovative surgical technique are reproducibility and safety. Understanding the learning curve of R-RPLND could benefit experienced robotic surgeons in their preparation for the critical surgical steps, which will ensure patient safety and oncologic efficacy [8]. In line with the current literature, our study indicates that R-RPLND is both safe and feasible in the primary and post-chemotherapy settings, and it is not inferior to open surgery in regard to short-term oncological outcomes [3,4,29,30].

The limitations of our study relate to its retrospective design and to the still-small number of cases for assessing the learning curve. Some selection bias may be associated with the fact that patients chosen for R-RPLND mostly had limited disease characteristics. Patients with extended disease or large volume retroperitoneal masses were subjected to open RPLND during the study period. The follow-up time for evaluating oncological outcomes is rather short, too.

To further identify how these learning curves affect training requirements for residents and fellows, a study examining junior staff is necessary to answer these questions further [8]. Generally, the learning curve in robotic surgery is multifaceted and influenced by factors such as the surgical procedure type, prior surgical experience, number of cases performed, and the metrics used to assess proficiency [31]. Surgeons generally experience rapid improvement in the initial cases, followed by a plateau as they gain more experience [32]. Understanding this learning curve is crucial for optimizing outcomes, enhancing surgical proficiency, and ensuring patient safety.

While the current data are encouraging, further research, especially prospective randomized trials, is needed to confirm these findings and assess the true efficacy and oncological safety of R-RPLND, as well as further investigation into optimal patient selection for R-RPLND. Specifically, long-term outcomes are currently still little understood. Continuous data collection and analysis are essential for refining patient selection criteria and optimizing surgical techniques for R-RPLND [11,12].

In addition, advancements in technology and training methodologies hold promise for shortening the learning curve of R-RPLND. Augmented reality (AR) and virtual reality (VR) platforms can enhance preoperative planning and intraoperative navigation, whereas artificial intelligence (AI) algorithms can provide real-time guidance and error prevention. Additionally, the development of standardized curricula and centralized databases for R-RPLND outcomes will enable more precise benchmarking and knowledge sharing.

In all, R-RPLND appears to be a safe and feasible alternative to open surgery for appropriately selected patients with GCTs [10,33].

## 5. Conclusions

R-RPLND for GCTs shows a clear learning curve, with significant advancement in OT, HS, and complication rates as surgeons gain experience. While early oncological outcomes are promising, further research is needed to confirm these findings and establish R-RPLND as a standard treatment option. The establishment of standardized curricula and centralized databases for R-RPLND is essential for precise benchmarking and effective knowledge sharing. This will lead to improved patient outcomes, enhanced research opportunities, and consistent care across medical centers. Overall, R-RPLND shows promising potential as a safe and effective treatment for GCTs, particularly when performed by experienced surgeons in specialized centers.

## Figures and Tables

**Table 1:** Clinical characteristics of the entire patient sample (*n* = 30).

Characteristics, *n* = 30	Median	IQR (Q1–Q3)
**Age (years)**	32.0	27.0–40.0
**BMI (kg/m^2^)**	25.75	23.9–27.4
**Preoperative LDH (U/L)**	193.5	159.5–226.8
**Preoperative AFP (µg/L)**	3.1	2.4–4.7
**Preoperative B-HCG (U/L)**	1,2	2.4–4.7
**Preoperative tumor mass (cm)**	2.0	1.2–3.0
**Laterality of primary tumor, *n* (%)**		
Right	16 (53.3)	
Left	13 (43.3)	
bilateral	1 (3.3)	
**Histology of primary tumor, *n* (%)**		
Non-seminoma	24 (80)	
Seminoma	6 (20)	
**Surgical indication, *n* (%)**		
Primary	10 (33.3)	
post-chemotherapy	13 (33.3)	
relapse	7 (23.3)	
**post-chemotherapy, *n* (%)**		
no	17 (56.6)	
yes	13 (43.3)	
**Prior abdominal operations, *n* (%)**		
no	26 (86.6)	
yes	4 (13.4)	
**Clinical stage (UICC) at time of RPLND, *n* (%)**		
I	1 (3.3)	
IIA	8 (26.6)	
IIB	18 (60.0)	
IIC	1 (3.3)	
**Prognostic group (IGCCCG), *n* (%)**		
Good prognosis	29 (96.6)	
Intermediate prognosis	1 (3.3)	

**Table 2:** Clinical preoperative characteristics of the three patient categories.

	Group A		Group B		Group C	
	Median	IQR	Median	IQR	Median	IQR
**Age (y)**	27.5	5.0	37.5	24.3	37.0	14.8
**BMI (kg/m^2^)**	26.1	3.3	27.2	2.9	25.9	6.1
**Preoperative LDH (U/L)**	244.0	107.0	246.0	76.0	172.5	43.5
**Preoperative AFP (µg/L)**	3.1	2.7	3.8	1.9	2.7	1.5
**Preoperative B-HCG (U/L)**	1.2	0.2	1.9	0.8	1.2	0.0
**size of retroperitoneal mass (cm)**	1.7	1.3	2.4	1.9	2.1	2.7
**post-chemotherapy, *n***						
no	8		5		5	
yes	2		5		5	
**Clinical stage (UICC) at time of RPLND, *n***						
I	0		1		0	
IIA	4		3		2	
IIB	6		5		8	
IIC	0		1		0	

**Table 3:** Clinical postoperative characteristics of the three patient categories.

	Group A		Group B		Group C	
	Median	IQR (Q1–Q3)	Median	IQR (Q1–Q3)	Median	IQR (Q1–Q3)
**OT (min)**	204	189–258	192	186–240	148	139–154
**HS (days)**	7	5–10	8	6.6–9.3	4	3.8–5
**EBL (mL)**	150	100–300	200	200–400	100	100–200
**Lymph Node Count (*n*)**	11	9–14	14.5	12–17	12	9–14
**Positive Lymph Nodes (*n*)**	1.5	0–3	1	0–4	3	1–4
**bowel recovery (days)**	4	3–4	4	3–4	2	2–3

**Table 4:** Association of operative time with clinical parameters—regression analysis.

	Univariate			Multivariate		
Characteristic	OR ^1^	95% CI ^1^	*p*-Value	OR ^1^	95% CI ^1^	*p*-Value
**Case Number**	0.9	0.8–0.9	**<0.001**	0.7	0.5–0.90	**0.01**
**post-chemotherapy**						
No	-	-		-	-	
Yes	1.1	0.7–1.6	0.8	0.8	0.1–10.6	0.9
**BMI**	0.9	0.9–1.1	0.8	0.9	0.7–1.2	0.7
**Preoperative tumor mass**	0.7	0.4–1.2	0.2	0.7	0.01–24.0	0.8
**Clinical stage**						
IIA	-	-		-	-	
IIB	0.9	0.6–1.4	0.6	1.0	0.1–68.1	0.6
IIC and III	0.9	0.5–1.7	0.7	1.1	0.02–150.0	0.8

^1^ OR = Odds Ratio, CI = Confidence Interval.

**Table 5:** Association of hospital stay with clinical parameters—regression analysis.

	Univariate			Multivariate		
Characteristic	OR ^1^	95% CI ^1^	*p*-Value	OR ^1^	95% CI ^1^	*p*-Value
**Case Number**	0.7	0.6–0.9	**0.04**	1.1	0.80–1.58	0.3
**post-chemotherapy**						
No	-	-		-	-	
Yes	3.1	0.3–31.1	0.6	0.09	0.002–4.1	0.1
**BMI**	0.8	0.6–0.9	0.3	0.8	0.6–1.2	0.2
**Prior abdominal operations**	21.8	1.3–345.9	0.3	107.5	1.2–9539.5	**0.04**
**EBL**	1.0	0.9–1.0	0.4	1.0	0.9–1.0	0.06
**Operative time**	1.0	0.9–1.0	0.2	1.0	0.9–1.1	0.1
**Overall complications (yes)**	2274.2	196.9–26,263.9	**<0.001**	4.6	0.08–244.3	0.3
**Clavien-Dindo I–II (yes)**	25,793.9	4086.6–162,805.6	**<0.001**	2958.3	148.5–58,919.1	**0.003**
**Clavien-Dindo III (yes)**	7.1	0.3–154.1	0.6	0.001	1.0–0.23	**0.02**

^1^ OR = Odds Ratio, CI = Confidence Interval.

**Table 6:** Association of lymph node yield with clinical parameters—regression analysis.

	Univariate			Multivariate		
Characteristic	OR ^1^	95% CI ^1^	*p*-Value	OR ^1^	95% CI ^1^	*p*-Value
**Case Number**	1.2	0.9–1.4	0.4	0.9	0.7–1.2	0.7
**post-chemotherapy**						
No	-	-		-	-	
Yes	0.03	<0.001–2.70	0.2	0.8	0.04–10.6	0.9
**BMI**	0.5	0.3–0.7	**0.001**	0.7	0.4–1.2	0.1
**Preoperative tumor mass**	3.7	0.7–19.2	0.2	43.3	6.5–287.2	**0.001**
**Clinical stage**						
IIA	-	-		-	-	
IIB	<0.001	<0.01–0.91	**0.05**	5.3	<0.001–0.005	**0.006**

^1^ OR = Odds Ratio, CI = Confidence Interval.

**Table 7:** Overview of postoperative complications of the three patient categories.

	Group A	Group B	Group C
**Clavien-Dindo I–II, *n***			
chylous ascites/leckage	3	2	0
blood transfusion	0	1	0
transient paresis of the legs	0	1	0
**Clavien-Dindo III–V, *n***			
aortic laceration and aortic grafting	0	1	0
vena cava laceration	0	0	1

## Data Availability

The original contributions presented in this study are included in the article. Further inquiries can be directed to the corresponding author(s).
